# Congenital Absence of Sternum: A Surgical Technique for Successful Outcome

**DOI:** 10.7759/cureus.54488

**Published:** 2024-02-19

**Authors:** Nishit Santoki, Sunder Negi, Snigdha Kumari, Anmol Bhatia, Rupesh Kumar

**Affiliations:** 1 Cardiothoracic and Vascular Surgery, Postgraduate Institute of Medical Education and Research, Chandigarh, IND; 2 Anesthesia and Intensive Care, Postgraduate Institute of Medical Education and Research, Chandigarh, IND; 3 Obstetrics and Gynecology, Postgraduate Institute of Medical Education and Research, Chandigarh, IND; 4 Radiology, Postgraduate Institute of Medical Education and Research, Chandigarh, IND

**Keywords:** pectus, sternum, thoracic defects, sternal defects, ectopia cordis

## Abstract

Congenital absence of sternum is a rare malformation of the anterior chest wall that needs surgical correction to avoid life-threatening complications as a consequence of such defect. It results from either partial or complete failure of fusion of mesenchymal strip during in utero organogenesis. The absence of sternum entails the risk of trauma to the mediastinal structures and other life-threatening complications. This defect is evaluated by a thorough clinical examination and computed tomography imaging of the thoracic cage to plan the surgical procedure. Early repair of the defect when the thoracic cage is still compliant yields the best result.

## Introduction

Congenital absence of sternum is a rare malformation of the anterior chest wall that results from either partial or complete failure of the fusion of mesenchymal strip during in utero organogenesis [1]. There are many syndromes associated with the defect like Cantrell’s pentalogy and PHACES (posterior fossa brain malformations, hemangiomas, arterial anomalies, coarctation of the aorta and cardiac defects, and eye abnormalities) syndrome [2]. Absent sternum entails the risk of injury to the mediastinal structures, hypothermia, increased insensible fluid losses, cyanosis due to altered compliance of the breathing pattern and recurrent infections of the chest if the overlying skin is exposed or gets exposed to the external environment after birth. Early surgical correction of defect should be considered as it becomes challenging as the babies grow older due to the lesser mobility of the rib cage, widening of the defect and the grown-up mediastinal structures in the lesser accommodating mediastinum [3]. Due to the rarity of the disease and the variation in the degree of the defect, the management protocols are confined only to some case reports. Few centers have described the use of repair of the sternal defect using synthetic mesh, porcine acellular dermal matrix, etc., but there remains a high risk of sternal infection and mediastinitis [4]. Early intervention is also preferred to support respiratory function and it is of utmost importance to maintain adequate preload, right heart function, ventilation and oxygenation to account for any alteration in lung compliance [5].

## Case presentation

We report a case of a 15-day-old baby delivered by caesarean section in a tertiary care hospital. She had normal growth milestones but on general examination was found to have a huge midline sternal defect with paradoxical breathing. The symptoms of respiratory distress aggravated due to a paradoxical suck in of the chest wall and bulging out of the anterior pericardium during cry and breathing, leading to repetitive spells of such episodes. The caloric consumption was suboptimal due to alteration in respiratory mechanics while feeding.

**Figure 1:**
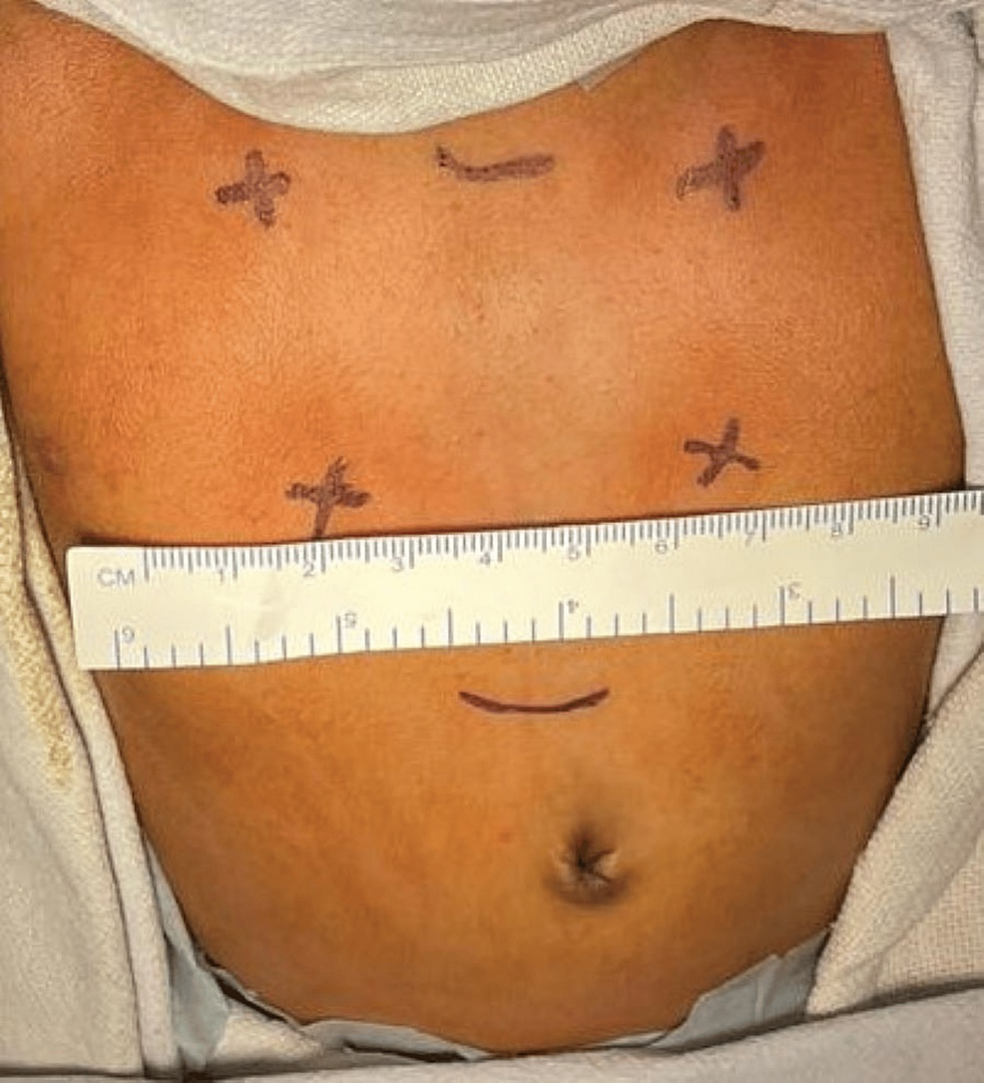
The maximum horizontal dimension of the defect at the level of nipple during expiration (45mm)

A two-dimensional echocardiogram was done to rule out any intra-cardiac defect; a non-contrast computer tomogram of thorax was done which revealed a complete absence of ossification centers of the sternal body and manubrium, a larger midline defect; covered by skin and subcutaneous tissue of maximum size 31x39.9mm extending from D1-D8 vertebral levels with depression of the anterior chest wall. The baby was taken to operation theatre on emergency basis in view of respiratory distress. After intubation the defect was measured and the maximum horizontal dimension of the defect at the level of nipple was 45mm (Figure 1).

**Figure 2:**
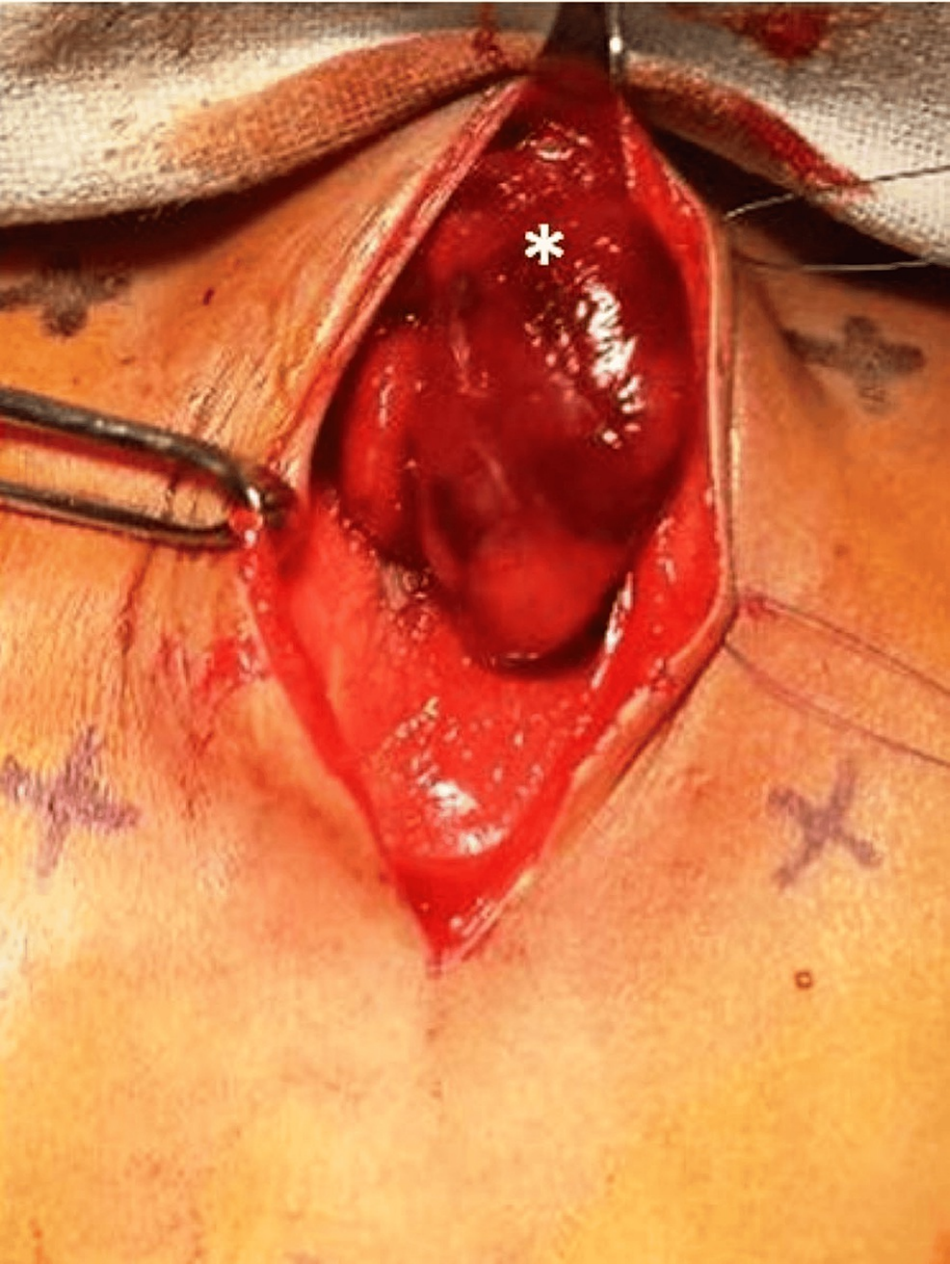
Excision of the thymus gland. (*Thymus gland)

A hypothetical suprasternal notch, xiphisternal end and edges of the floating ribs were marked for symmetrical apposition of the defect. The skin over the defect was incised taking care not to open the pericardium lying just beneath the skin. This will prevent unnecessary exposure of the heart and the large vessels to the surroundings and hence reduce the risk of pericarditis. The skin was mobilized all throughout the defect. The edges of the ribs were delineated. Both lobes of the thymus were excised to avoid any compression over the cardiac chambers and hence the hemodynamic compromise (Figure 2). Bilateral longitudinal pleurotomy was performed to further give some additional space to the heart.

**Figure 3:**
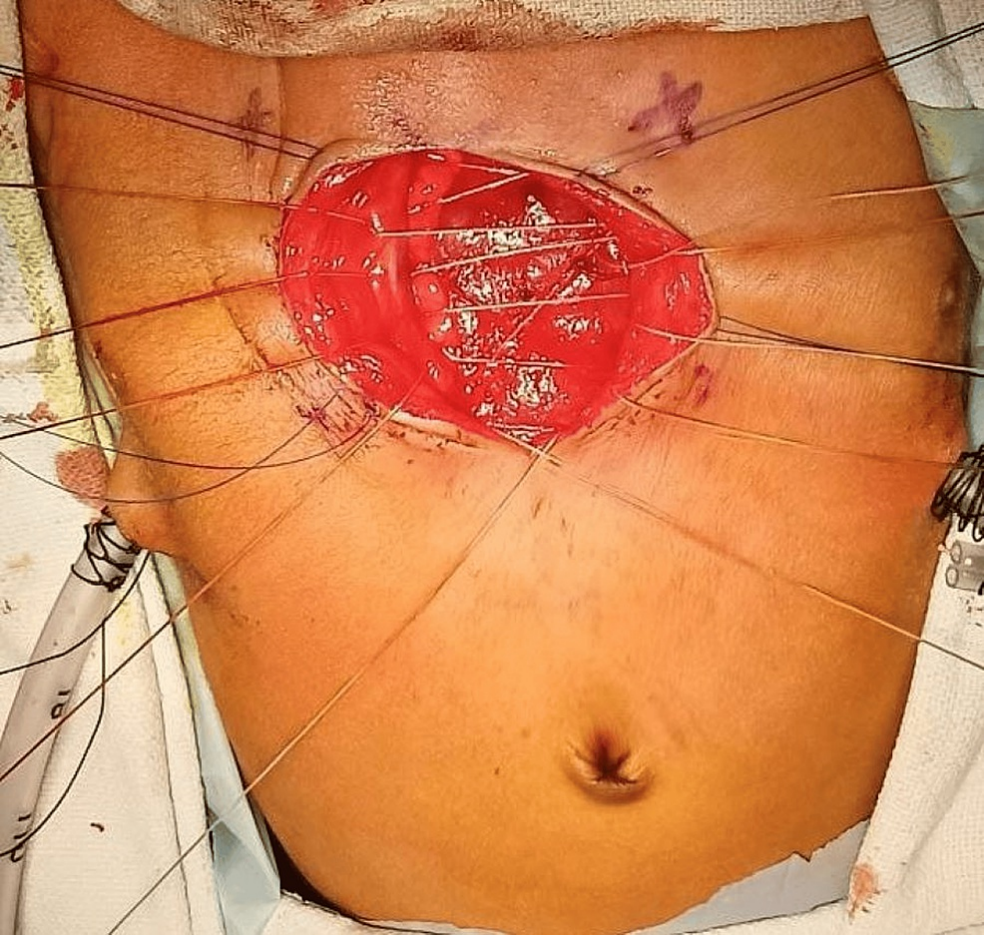
Edges of the ribs brought closer with a non-absorbable suture

**Figure 4:**
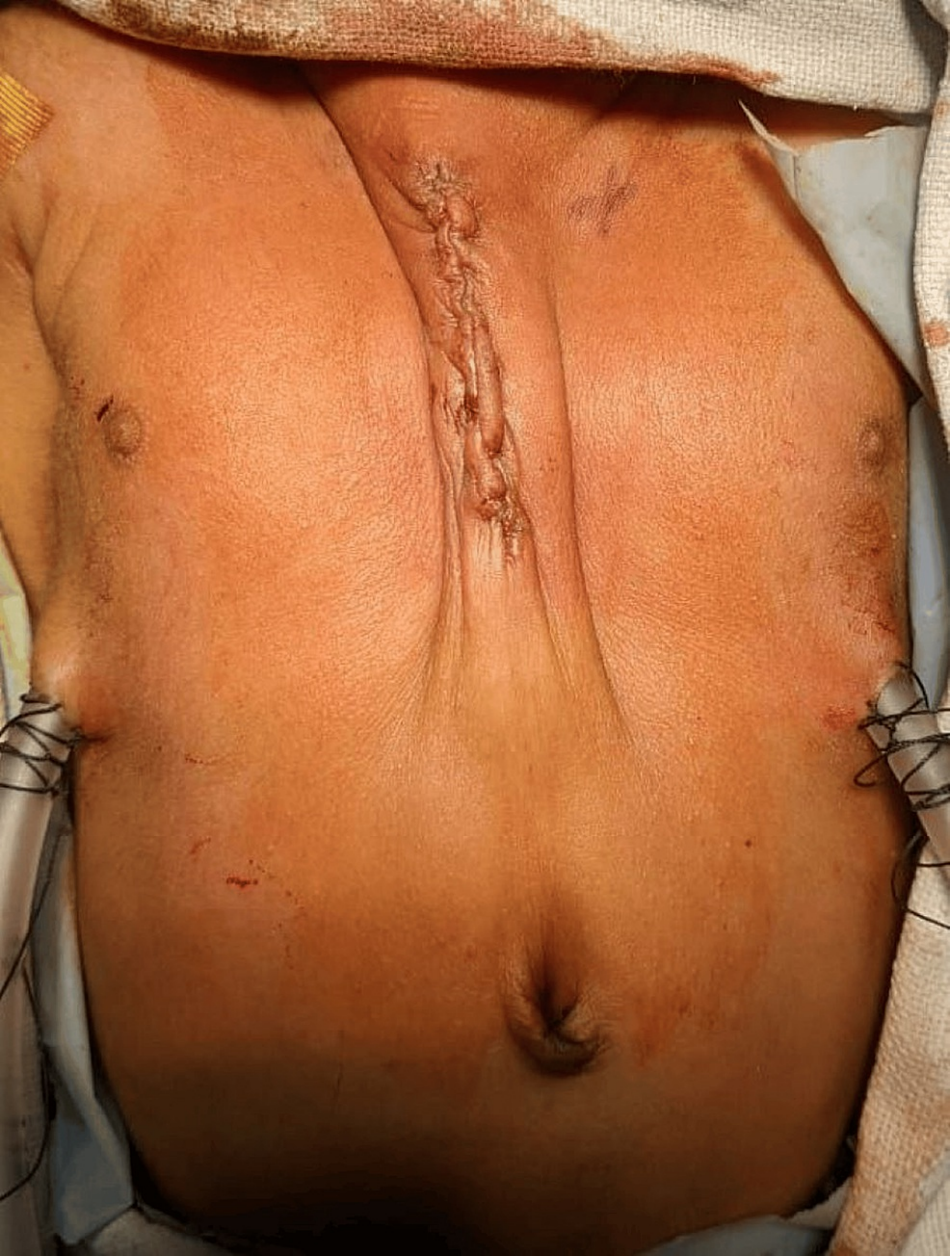
Wound after closure of the defect.

The edges of the ribs were brought closer with multiple non-absorbable sutures (Figure 3). The suture used for closure of the sternal defect was 2-0 Ethibond suture which is a non-absorbable suture and is made from braided polyester coated with polybutylate for easier tying. There was a slight increase in heart rate and central venous pressure but the arterial blood pressure was maintained. With 15 degree reverse Trendlenberg tilt of the operating table, the hemodynamic settled to its normal and after observing for 10 minutes, the sutures were finally tied together. The subcutaneous tissues were also approximated together. The excess skin was not excised as if the procedure needs to be reversed there will be a skin to cover the defect. Baby was extubated 48 hours after surgery. The baby was discharged on the seventh post-operative day with satisfactory outcome (Figure 4).

## Discussion

Congenital absence of sternum is a rare malformation. Due to the rarity of the disease whatever clinical experiences have been achieved with the management strategies should be shared among the clinicians. It necessitates intervention in neonatal periods when the chest wall is still pliable. Computer tomogram of thorax helps in planning the closure of the defect. Nasogastric tube insertion is mandatory as it avoids any upper abdomen distention and hence respiratory distress. Transesophageal probe insertion should be avoided as it might consume some space in the mediastinal cavity. Midline skin incision should be made very meticulously to avoid opening of the pericardium and any inadvertent injury to the cardiac structures. The clavicles should not be mobilized as approximation of the ends of clavicle will lead to a rise in the jugular venous pressure and distension of neck veins further leading to hypotension and hemodynamic compromise. After full mobilization of the defect the edges of the ribs should be approximated with the opposite ends with non-absorbable sutures. Thymectomy and bilateral pleutotomy with insertion of bilateral intercostal chest tubes is a must to accommodate the mediastinal structures without any compression. Momentarily changes in the hemodynamics should not discourage the procedure of primary closure of the defect as the hemodynamics gradually reverts back to its normal. The anesthetic goals in this scenario mandate maintaining adequate preload and right heart function as well as manipulating ventilation and oxygenation to account for any alteration in lung compliance.

## Conclusions

Congenital absence of sternum is a rare malformation. It necessitates intervention in neonatal periods when the chest wall is still pliable. Primary closure without any graft/prosthetic material should be highly encouraged.
